# Etude de l'utilisation des médicaments chez les enfants dans un contexte de gratuité des soins

**DOI:** 10.11604/pamj.2019.34.194.19613

**Published:** 2019-12-12

**Authors:** Boukary Sana, Ahmed Kaboré, Hervé Hien, Brice Evance Zoungrana, Nicolas Meda

**Affiliations:** 1 Direction Générale de l'Accès aux Produits de Santé, Ouagadougou, Burkina Faso; 2 Université Joseph Ki Zerbo, Département de Santé Publique, Ouagadougou, Burkina Faso; 3 Institut National de Santé Publique, Ouagadougou, Burkina Faso; 4 Institut de Recherche en Science de la Santé, Bobo-Dioulasso, Burkina Faso

**Keywords:** Usage rationnel des médicaments, gratuité des soins, enfants de 0 à 5 ans, Rational use of medicines, free health care, children aged 0 to 5 years

## Abstract

**Introduction:**

La rationalisation de la prescription des médicaments est une préoccupation pour les politiques et systèmes de santé des pays africains. L'objectif de cette étude était d'analyser l'utilisation des médicaments chez les enfants de 0 à 5 ans dans un contexte de gratuité des soins.

**Méthodes:**

Il s'agissait d'une étude transversale sur l'utilisation des médicaments, réalisée dans 20 centres de santé du 1^er^ niveau de contact à Ouagadougou, sélectionnés de façon aléatoire. Le référentiel de l'Organisation Mondiale de la Santé (OMS) et du Réseau Internationale pour l'usage rational des médicaments (OMS/INRUD) a été utilisé pour l'analyse des données. Des statistiques descriptives ont été utilisées sous forme de moyenne et écart type. Les différences ont été mesurées à l'aide d'un test ANOVA.

**Résultats:**

Au total, 1206 prescriptions entre avril 2016 et mars 2017 ont été examinées. Le nombre de médicaments prescrits par ordonnance était de 2,9, le pourcentage de médicaments prescrits par nom générique était de 88,7% et 97,7% des médicaments prescrits étaient inscrits sur la Liste Nationale des Médicaments Essentiels (LNMCE). Le pourcentage de consultations ayant donné lieu à la prescription d'antibiotiques était de 83,2%, et 9,3% des prescriptions contenait au moins un produit injectable.

**Conclusion:**

La prescription irrationnelle concerne surtout l'utilisation des antibiotiques. Une vigilance particulière doit être faite au cours des soins des enfants de moins de 5 ans pour éviter la surconsommation des médicaments et l'émergence des résistances liées à l'utilisation des antibiotiques.

## Abstract (en)

**Introduction:**

Rational medical prescription is a concern for the health systems and policies of African countries. The purpose of this study was to analyze the use of medicines in children aged 0 to 5 years receiving free care.

**Methods:**

We conducted a cross-sectional study considering the use of medicines in 20 randomly selected health facilities level 1 in Ouagadougou. The reference of the World Health Organization and of the International Network for the Rational Use of Drugs (WHO/INRUD) was used to analyze data. Descriptive statistics were used as the average and standard deviation. Differences were measured using ANOVA test.

**Results:**

In total, 1.206 prescriptions were reviewed between April 2016 and March 2017. The number of medicines issued on prescription was 2.9, the percentage of generic medicines prescribed was 88.7% and 97.7% of medicines prescribed were registered on the National List of Essential Medicines. The percentage of consultations during which antibiotics were prescribed was 83.2%, and 9.3% of prescriptions contained at least one injectable product.

**Conclusion:**

Irrational prescription is mainly relates to the use of antibiotics. Essential alertness should be given to the treatment of children less than 5 years to avoid an excessive consumption of medicines and the emergence of antibiotic resistance.

## Introduction

La rationalisation des prescriptions des médicaments est un problème majeur dans les systèmes de santé des pays en développement [1, 2]. Au cours des dernières décennies, de nombreuses études ont montré que les pratiques de prescriptions sont peu respectueuses des normes en vigueur [3]. Ainsi l'on observe en Afrique subsaharienne une tendance à la sur-prescription des médicaments par ordonnance qui varie d'un pays à l'autre. Il est de 4,3 au Ghana; 3,9 au Nigeria; 3,0 au Cameroun et de 2,4 au Burkina Faso [1, 4-6]. Cette utilisation non rationnelle des médicaments entraine des dépenses en importations atteignant 80% des coûts en santé [7, 8]. Depuis les années 1980, presque tous les pays africains ont instauré le paiement direct des soins par les patients lorsqu'ils utilisent les services de santé [9], ainsi les programmes de recouvrement des coûts des soins de santé primaires se sont multipliés et ont permis une meilleure disponibilité des médicaments essentiels génériques dans les centres de santé [7, 10, 11]. Cependant de nombreuses études ont montré que le mode de paiement direct exclut les populations vulnérables de l'accès aux services de santé [4, 12, 13]. Pour permettre un accès équitable, surtout des populations vulnérables aux services de santé, certains pays africains ont abolis le paiement direct pour des catégories de personnes ou de services facilement identifiables [14, 15]. Certes l'abolition de payement semble lever l'obstacle de la barrière financière mais les décideurs se posent toujours des questions sur les meilleures pratiques de gestion des systèmes d'exemption de paiement des soins, notamment la disponibilité des médicaments [9, 16], car tous les pays qui ont expérimenté l'exemption de paiement des soins en Afrique, ont connu des problèmes de disponibilité, et de gestion des médicaments. On notait une distribution tardive des médicaments à Madagascar, les agents de santé ghanéens ont apprécié une meilleure disponibilité des fonds pour l'achat de médicaments au début de la politique de gratuité, mais cette situation n'a pas duré [13, 16]. En Ouganda, les ruptures de stocks de médicaments ont augmenté, après une année de mise en œuvre de l'exemption des soins. Au Burkina Faso, en Côte d'Ivoire et au Mali on avait constaté une rupture de médicaments dans les dépôts pharmaceutiques dans les formations sanitaires obligeant les bénéficiaires à acheter des médicaments dans les officines pharmaceutiques privées. Au Niger, le constat était peu reluisant avec une difficulté de la centrale d'achat et de distribution des médicaments à approvisionner adéquatement les formations sanitaires, la hausse de l'utilisation des médicaments par consultation qui était 3,4 serait la cause des ruptures de stocks en médicaments essentiels et la fermeture de certaines formations sanitaires pour faute de stock de médicaments [14, 16, 17]. En avril 2016, le Burkina Faso a adopté les mesures de gratuité des soins notamment au profit de l'enfant de 0 à 5 ans et de la femme. C'est dans ce contexte que cette étude a été initiée pour analyser l'utilisation des médicaments au 1^er^ niveau de contact de soins à Ouagadougou au Burkina Faso.

## Méthodes

**Type d'étude:** il s'agissait d'une étude transversale et les indicateurs d'utilisation des médicaments de l'OMS/INRUD ont été utilisés [11, 18]. La collecte des données s'est déroulée du 16 au 30 avril 2017.

**Cadre de l'étude:** l'étude a été réalisée au niveau des centres de santé de 1^er^ niveau des soins (centres de santé et promotion sociale et centres médicales urbains) à Ouagadougou.

**Echantillonnage et taille de la population d'étude:** dans le cadre d'une enquête transversale de la prescription rationnelle des médicaments dans une zone (ville, district ou région), il faut étudier au moins 600 consultations et un échantillon de 20 centre de santé soit au moins 30 consultations par centre de santé [18, 19]. Conforment à la recommandation ci-dessus, l'étude a porté sur 20 centres de santé choisis de façon aléatoire sur la base de la liste des 51 centres de santé de premier niveau de contact des soins de la ville de Ouagadougou. Au niveau des centres de santé retenus, l'étude a porté sur les pratiques de prescription de 1200 consultations soit un échantillon de 60 ordonnances par formation sélectionnée au cours d'une année de mise en œuvre de la gratuité des soins: du 02 avril 2016 au 01 avril 2017. L'unité d'observation était l'ordonnance figurant dans les registres de consultation. Elle portait sur les médicaments prescrits chez les enfants de 0 à de 5 ans admis en consultation externe dans la cadre de la gratuité des soins. L'échantillonnage des ordonnances a consisté à un tirage systématique de cinq ordonnances chaque mois soit un total de cent ordonnances par mois. Les cinq ordonnances ont été choisies par pas de tirage en fonction de l'effectif mensuel des ordonnances.

**Technique et outils de collecte:** les fiches de collecte ont été utilisées pour recueillir sur les ordonnances échantillonnées. Les caractéristiques des données recueillies sur les ordonnances échantillonnées étaient les suivantes: l'âge de l'enfant, la date de la consultation, le nombre total de médicaments prescrits, le nombre de médicaments prescrits par nom générique, la présence ou non d'antibiotique, présence ou non de produits injectables, le coût des médicaments, le coût de l'ordonnance.

**Analyse des données:** les données ont été traitées et analysées à l'aide du logiciel Epi Info TM 7.2. Le référentiel de l'OMS/INRUD a été utilisé pour l'analyse des données: 1) nombre moyen de médicaments prescrits par ordonnance (≤ 2); 2) pourcentage de médicaments prescrits par nom générique (100%); 3) pourcentage de prescription avec au moins un antibiotique (≤ 30%); 4) pourcentage de prescription avec au moins un produit injectable (≤ 20%); 5) pourcentage de médicaments inscrits sur la liste nationale (100%); 6) pourcentage des ordonnances complètement servies (100%). Des statistiques descriptives ont été utilisées sous forme de moyenne, médiane et écart type. La différence entre les centres de santé et les périodes ont été mesurées à l'aide d'un test ANOVA.

## Résultats

Nous avons collecté 1206 ordonnances dans les vingt (20) Centres de Santé de Ouagadougou. Le nombre moyen de médicaments prescrits par ordonnance était de 2,9 et ce nombre a varié de 2,1 à 3,5 dans les centres de santé (Tableau 1), il est resté stable durant la 1^ère^ année de mise en œuvre (p = 0,21) (Tableau 2). Le pourcentage de médicaments prescrits par nom générique était de 88,7%. Quant au pourcentage de médicaments inscrits sur la liste nationale de médicaments essentiels, il était de 97,7%. Chez les enfants venus en consultation, 83,2% des prescriptions contenaient au moins un antibiotique, les antibiotiques les plus prescrits étaient le cotrimoxazole (48%), l'amoxicilline (26%) et l'érythromycine (8%). En effet sur les 61 médicaments qui ont connus au moins une fois la rupture dans les centres de santé visités, la moitié était des antibiotiques. Quant à la prescription des produits injectables, 9,3% des prescriptions contenaient au moins un produit injectable. Le pourcentage d'ordonnances complètement servies était de 63,0% (Tableau 1), et durant la première année de mise œuvre, il a été constaté une diminution progressive du pourcentage d'ordonnances complètement servies, passant de 80% au mois d'avril 2016 à 54% au mois de mars 2017 (Tableau 2). Le coût moyen des médicaments par ordonnance était 1.30 US$ (XOF 768). Et quant à son évolution durant une année, le constat observé était une baisse, passant de 1.65 US$ (XOF 974) à 1.07 US$ (XOF 629) soit une diminution d'un tiers (p ≤ 0,0001) (Tableau 2).

**Tableau 1:** Indicateurs de prescription OMS/INRUD pendant une année mise en œuvre de la gratuité des soins à Ouagadougou, Burkina Faso

Centres de santé	Nombre moyen de medicaments Prescrits par ordonnance (IC à 95 %)	Pourcentage de médicaments prescrits par nom générique (IC à 95 %)	Pourcentage de médicaments inscrits sur la liste nationale de médicaments essentiels. (IC à 95 %)	Pourcentage de prescriptions avec un antibiotique (IC à 95 %)	Pourcentage de prescriptions avec un produit injectable (IC à 95 %)	Coût médian des médicaments par ordonnance (US$). (IC à 95 %)	Pourcentage des ordonnances complément servies (IC à 95 %)
1	3,2 (2,9 - 3,5)	93,0 (89,6 - 96,4)	100	71,7 (58,5 - 82,5)	8,3 (2,8 - 18,4)	1,87 (1,60 - 2,15)	71,7 (58,6 - 82,6)
2	2,9 (2,6 - 3,1)	76,6 (71,9 - 81,4)	97,7 (95,1 - 100)	81,3 (69,1 - 90,3)	3,4 (0,4 - 11,7)	1,66 (1,43 - 1,88)	62,7 (49,1 - 74,9)
3	3,3 (2,9 - 3,6)	81,3 (76,3 - 86,3)	99,0 (97,5 - 100)	83,3 (71,4 - 91,7)	25,0 (14,7 - 37,9)	1,22 (0,99 - 1,46)	60,0 (46,5 - 72,4)
4	2,9 (2,6 - 3,1)	96,5 (93,8 - 99,1)	100	76,3 (63,4 - 86,4)	0	1,49 (1,26 - 1,72)	55,9 (42,4 - 68,8)
5	2,7 (2,4 - 3,0)	94,6 (91,7 - 97,5)	98,9 (97,4 - 100)	85,5 (74,2 - 93,1)	11,3 (4,6 - 21,9)	1,50 (1,22 - 1,78)	53,2 (40,1 - 66,0)
6	2,8 (2,5 - 3,1)	88,9 (83,5 - 94,2)	98,0 (94,6 - 100)	78,7 (66,3 - 88,1)	0	1,46 (1,21 -1,72)	59,0 (45,7 - 71,4)
7	2,7 (2,4 - 3,0)	79,4 (72,2 - 86,5)	88,2 (82,4 - 93,9)	73,3 (60,3 - 83,9)	20,0 (10,8 - 32,3)	1,13 (0,90 - 1,36)	41,7 (29,1 - 55,1)
8	3,0 (2,7 - 3,2)	79,3 (74,5 - 84,0)	88,7 (84,3 - 93,1)	88,3 (77,4 - 95,1)	1,7 (0,1 - 8,9)	0,92 (0,74 - 1,11)	35,0 (23,1 - 48,4)
9	2,4 (2,1 - 2,6)	95,8 (92,6 - 98,9)	100	72,1 (59,2 - 82,8)	13,1 (5,8 - 24,2)	0,93 (0,72 - 1,13)	95,1 (86,3 - 98,9)
10	3,4 (3,1 - 3,7)	82,9 (77,9 - 87,9)	96,9 (94,6 - 99,0)	90,1 (79,8 - 96,3)	16,4 (8,1 - 28,1)	1,25 (1,04 - 1,46)	50,8 (37,7 - 63,7)
11	2,9 (2,6 - 3,1)	86,2 (81,0 - 91,4)	92,4 (88,2 - 96,7)	96,7 (88,4 - 99,5)	5,0 (1,0 - 13,9)	0,73 (0,59 - 0,86)	35,0 (23,1 - 48,4)
12	2,9 (2,7 - 3,1)	82,9 (77,8 - 88,1)	98,1 (95,9 - 100)	90,1 (79,8 - 96,3)	9,8 (3,7 - 20,2)	1,33 (1,10 - 1,56)	50,8 (37,7 - 63,8)
13	2,7 (2,4 - 3,0)	79,2 (73,7 - 84,7)	98,1 (95,8 - 100)	86,7 (75,4 - 94,1)	0	1,42 (1,22 - 1,61)	83,3 (71,4 - 91,7)
14	2,9 (2,6 - 3,1)	96,2 (93,0 - 99,3)	99,5 (98,4 - 100)	91,8 (81,9 - 97,3)	1,64 (0,1 - 08,8)	1,07 (0,97 - 1,17)	86,9 (75,9 - 94,1)
15	2,9 (2,6 - 3,1)	96,0 (93,3 - 98,7)	99,0 (97,6 - 100)	83,3 (71,4 - 91,7)	16,7 (8,3 - 28,5)	1,18 (1,02 - 1,35)	63,3 (50,0 - 75,4)
16	2,1 (1,9 - 2,2)	95,5 (91,9 - 99,1)	100	60,0 (46,5 - 72,4)	0	0,97 (0,82 - 1,09)	98,3 (91,1-99,9)
17	2,9 (2,6 - 3,2)	90,1 (85,2 - 95,0)	100	84,7 (73,0 - 92,8)	20,3 (10,9 - 32,8)	1,01 (0,85 - 1,17)	64,4 (50,9 - 76,4)
18	3,2 (2,9 - 3,4)	89,7 (85,5 - 93,9)	100	88,3 (77,4 - 95,1)	5,0 (1,0 - 13,9)	1,29 (1,11 - 1,47)	48,3 (35,2 - 61,6)
19	3,5 (3,2 - 3,8)	98,5 (97,0 - 100)	100	86,7 (75,4 - 94,1)	26,7 (16,1 - 39,7)	1,59 (1,37 - 1,81)	58,3 (44,9 - 70,9)
20	2,9 (2,7 - 3,1)	92,2 (88,5 - 95,9)	100	95,1 (86,5 - 99,0)	3,2 (0,4 - 11,2)	1,98 (1,79 - 2,17)	85,5 (74,2 - 93,1)
Total	2,9 (2,8 - 3,0)	88,7 (87,7 - 89,8)	97,7 (97,2 - 98,3)	83,2 (81,0 - 85,2)	9,3 (7,8 - 11,2)	1,30 (1,25 - 1,35)	63,0 (60,2 - 65,7)
(ANOVA)	p ≤ 0,0001	p ≤ 0,0001	p ≤ 0,0001	p ≤ 0,0001	p ≤ 0,0001	p ≤ 0,0001	p ≤ 0,0001

**Tableau 2:** Évolution des indicateurs de prescription OMS/INRUD pendant une année à Ouagadougou, Burkina Faso

Période (Mois)	Nombre moyen de medicaments Prescrits par ordonnance (IC à 95 %)	Coût médian des médicaments par ordonnance (US$) (IC à 95 %)	Pourcentage des ordonnances complément servies (IC à 95 %)
**Avril 2016**	2,9 (2,7 - 3,1)	1,65 (1,51 - 1,79)	80
**Mai 2016**	2,7 (2,5 - 2,8)	1,41 (1,27 - 1,54)	78
**Juin 2016**	2,7 (2,4 - 2,9)	1,51 (1,31 - 1,71)	78
**Juillet 2016**	2,8 (2,5 - 3,0)	1,43 (1,24 - 1,60)	71
**Aout 2016**	3,0 (2,8 - 3,1)	1,24 (1,10 - 1,38)	68
**Septembre 2016**	2,9 (2,6 - 3,1)	1,28 (1,08 - 1,31)	61
**Octobre 2016**	3,1 (2,8 - 3,3)	1,31 (1,14 - 1,48)	52
**Novembre 2016**	2,9 (2,7 - 3,1)	1,30 (1,13 - 1,47)	53
**Décembre 2016**	3,1 (2,9 - 3,3)	1,26 (1,10 - 1,43)	54
**Janvier 2017**	2,9 (2,7 - 3,1)	1,02 (0,86 - 1,17)	49
**Févier 2017**	2,9 (2,7 - 3,1)	1,12 (0,95 - 1,28)	58
**Mars 2017**	3,0 (2,7 - 3,2)	1,07 (0,91 - 1,22)	54
**Total**	**2,9 (2,8 - 3,0)**	**1,30 (1,25 - 1,35)**	**63,0 (60,2 - 65,7)**
**ANOVA**	**p = 0,21**	**p ≤ 0,0001**	**p ≤ 0,0001**

## Discussion

Dans cette étude, ce sont les indicateurs de l'utilisation des médicaments de l'OMS/INRUD qui ont été utilisés pour décrire les pratiques de l'utilisation des médicaments actuelles par rapport aux normes établies par l'OMS [11]. Ces indicateurs peuvent être une source d'informations de base pour un suivi continu dans un contexte d'exemption des soins.

**La prescription des médicaments:** notre étude a montré un nombre moyen de médicaments prescrits par ordonnance de 2,9 à Ouagadougou, ce qui était supérieur à la norme OMS (≤ 2). Ce constat pourrait se justifié par le niveau de la formation initiale des agents de santé et le non-respect des protocoles et guides thérapeutiques. Car dans les pays en développement l'enseignement ne correspond pas toujours aux protocoles et guides thérapeutiques nationaux en vigueur [4, 20, 21]. En effet une utilisation non-rationnelle des médicaments peut stimuler une demande inappropriée de la part des bénéficiaires et une surutilisation des médicaments pouvant occasionner des ruptures dans les centres de santé [11]. Ce constat des ruptures de médicaments dans les Centres de Santé à Ouagadougou était similaire aux constats rapportés, des pays Africains qui ont expérimenté la gratuité des soins [9, 16]. Les ruptures de médicament pourraient entrainer une perte de confiance des bénéficiaires vis-à-vis de la politique de gratuité et engendrer des problèmes de morbidité et de mortalité, plus particulièrement chez les enfants [4, 9]. Notre étude a montré également que le pourcentage de médicaments prescrits par nom générique a atteint 88,7%, Ce pourcentage était inférieur à celui recommandé par l'OMS (100%). Ce résultat pourrait s'expliqué par la disponibilité des médicaments essentiels génériques au niveau des centres de santé publics avec le système de recouvrement coût [22-24].

**Utilisation des antibiotiques:** notre étude a montré une prescription des antibiotiques chez 83% des enfants ayant consulté dans les centres de santé de 1^er^ niveau de contact. Dans cette étude, il n'était pas question de faire la différence entre une prescription antibiotique appropriée et inappropriée, mais de voir la prescription irrationnelle qui peut être un problème de santé publique dans notre contexte. Ce pourcentage de prescription des antibiotiques était supérieur à la norme OMS (≤ 30%). Les statistiques sanitaires de la région du centre de 2016 et 2017, montraient que les infections bactériennes représentaient moins de 30% des motifs de consultations externes chez les enfants [25]. Cette étude a prouvé que les prescripteurs des centres de santé de 1^er^ niveau de contact des soins ne tiennent pas compte de leur diagnostic avant de prescrire les antibiotiques chez les enfants de 0 à 5 ans. L'absensce de laboratoires de microbiologie dans les centres de santé primaires des pays Africains amènent les prescripteurs à diagnostiquer les infections bactérienne sur un jugement clinique [6,26, 27]. Avec la mesure de la gratuité des soins, en utilisant des antibiotiques chez plus d'enfants venus en consultation, cela pourrait entrainer des résistances aux antibiotiques.

**Utilisation des injectables:** notre étude a montré une prescription des produits injectables chez 9,3% des enfants venus en consultation externe dans les centres de santé de 1^er^ niveau de contact de Ouagadougou. Ce faible taux par rapport à la norme OMS (≤ 20%), pourrait s'expliquer par le fait que le cout d'une injection est toujours supérieur à celui d'un traitement par voie orale et la préférence de la voie orale par les parents des enfants [8, 28, 29].

## Conclusion

Notre étude a analysé les pratiques de l'utilisation des médicaments chez les enfants de 0 à 5 ans au 1^er^ niveau de soins de la ville de Ouagadougou, dans un contexte de la gratuité des soins au Burkina Faso, en utilisant les indicateurs de prescription de médicaments de l'OMS/INRUD. Les résultats ont montré que les indicateurs de l'utilisation des médicaments ne respectent pas les normes OMS, cela traduit la nécessité de mener une réflexion sur le rôle et le profil de prescripteurs au Burkina Faso. Les médicaments représentent plus de 2/3 des dépenses des soins dans le cadre de la gratuité, il est nécessaire de promouvoir une utilisation rationnelle pour la pérennisation des politiques de gratuité des soins.

### Etat des connaissances actuelles sur le sujet

- La rationalisation des prescriptions des médicaments est un problème majeur dans les pays en développement;
- L'augmentation de la résistance aux antibiotiques;
- La mise en œuvre des programmes de gratuité des soins sont confrontés à des ruptures de médicaments.

### Contribution de notre étude à la connaissance

- Le non-respect des protocoles et guides thérapeutiques standards;
- La prescription systématique des antibiotiques avec la mesure de la gratuité des soins;
- La baisse de la disponibilité des médicaments avec la mesure de la gratuité des soins.

## Conflits d’intérêts

Les auteurs ne déclarent aucun conflit d’intérêts.
